# A critical role for the ATP-sensitive potassium channel subunit K_IR_6.1 in the control of cerebral blood flow

**DOI:** 10.1177/0271678X18780602

**Published:** 2018-06-04

**Authors:** Patrick S Hosford, Isabel N Christie, Arun Niranjan, Qadeer Aziz, Naomi Anderson, Richard Ang, Mark F Lythgoe, Jack A Wells, Andrew Tinker, Alexander V Gourine

**Affiliations:** 1 William Harvey Research Institute, Barts and the London School of Medicine and Dentistry, London, UK; 2 Centre for Cardiovascular and Metabolic Neuroscience, Neuroscience, Physiology & Pharmacology, University College London, London, UK; 3 UCL Centre for Advanced Biomedical Imaging, Division of Medicine, University College London, London, UK

**Keywords:** Cerebral blood flow, cerebrovascular reactivity, functional magnetic resonance imaging, hypoxia, neurovascular coupling

## Abstract

K_IR_6.1 (KCNJ8) is a subunit of ATP sensitive potassium channel (K_ATP_) that plays an important role in the control of peripheral vascular tone and is highly expressed in brain contractile cells (vascular smooth muscle cells and pericytes). This study determined the effect of global deletion of the K_IR_6.1 subunit on cerebral blood flow, neurovascular coupling and cerebral oxygenation in mice. In K_IR_6.1 deficient mice resting cerebral blood flow and brain parenchymal partial pressure of oxygen (*P*O_2_) were found to be markedly lower compared to that in their wildtype littermates. However, cortical blood oxygen level dependent responses triggered by visual stimuli were not affected in conditions of K_IR_6.1 deficiency. These data suggest that K_ATP_ channels containing K_IR_6.1 subunit are critically important for the maintenance of normal cerebral perfusion and parenchymal *P*O_2_ but play no significant role in the mechanisms underlying functional changes in brain blood flow.

## Introduction

Neurovascular coupling dynamically regulates the blood supply to active brain areas to match metabolic demand and supply.^1^ In pathological conditions, including Alzheimer’s disease,^2^ hypertension,^3^ stroke,^4^ traumatic brain injury^5^ and glioma,^6^ compromised cerebral blood flow (CBF) may contribute to the development and/or progression of the disease, highlighting the importance of understanding the mechanisms controlling resting and dynamic CBF.

Ion channels that determine the membrane potential of cerebrovascular smooth muscle cells (potassium channels for example) are likely to be important for the control of CBF (for review see Longden et al.^7^). Even small increases in extracellular potassium can have a profound effect on vascular tone by activating inwardly rectifying potassium channels expressed in the vascular smooth muscle cells.^8,9^ Recent data suggest that K_IR_2.1 potassium channels mediate the effect of increased extracellular K^+^ on cerebral vasculature and contribute to the operation of mechanisms underlying increases in local CBF which follow changes in neuronal activity.^10^

Another notable family of K^+^ channels include ATP-sensitive potassium channels (K_ATP_). In the periphery, K_IR_6.1 plays an important role in determining resting vascular tone, peripheral resistance and, therefore, systemic arterial blood pressure. Global K_IR_6.1 deficiency in an animal (mouse) model was reported to be associated with chronically elevated (by ∼20 mmHg) systemic arterial blood pressure.^11^ In the brain, drugs which promote opening of K_ATP_ channels were reported to induce dilations of basilar and middle cerebral arteries in a rat model.^12^

K_IR_6.1 is also abundantly expressed by brain pericytes and was previously suggested to be used as a molecular marker of this cell type.^13^ Evidence is accumulating that these contractile cells play a critical role in neurovascular coupling,^14,15^ although this idea has been disputed.^16,17^ K_ATP_ channel activation hyperpolarises pericytes as demonstrated in retinal microvasculature.^18^ As K_ATP_ channels are sensitive to changes in intracellular concentrations of ATP/ADP, this positions K_IR_6.1 as a possible metabolic sensor of pericytes.

In this study, we determined the effect of global deletion of the K_IR_6.1 channel on resting CBF, cerebrovascular reactivity to CO_2_, neurovascular coupling and brain tissue *P*O_2_ in mice. The data obtained suggest that K_IR_6.1 is critically important for the maintenance of normal cerebral perfusion and oxygenation but plays no significant role in the generation of blood oxygen level dependent (BOLD) responses to sensory stimulation.

## Materials and methods

### Animals

The experiments were performed on 35 male mice (two to three months old) in accordance with the European Commission Directive 86/609/EEC (European Convention for the Protection of Vertebrate Animals used for Experimental and Other Scientific Purposes) and the United Kingdom Home Office (Scientific Procedures) Act (1986) with project approval from the Institutional Animal Care and Use Committee. The results of the animal experimentations are reported in accordance with ARRIVE guidelines.

Generation of the K_IR_6.1 knockout mouse strain has been previously described in detail.^11^ Briefly, crossing homozygous K_IR_6.1 floxed mice with a mouse ubiquitously expressing Cre recombinase on a C57Bl-backgound produced mice with global deletion of one allele of K_IR_6.1. The progeny were then backcrossed onto a C57Bl/6 background for at least six generations. Resulting K_IR_6.1^+/−^ mice were then crossed to produce homozygotes.

The animals were housed in a temperature controlled room at 21 ± 2℃ and a relative humidity of 55 ± 10%, with a 12-h light/12-h dark cycle with a 30 min twilight period.

### fMRI and analysis

All MRI experiments were performed using a 9.4T MRI scanner (Agilent Inc.), a 72 mm inner diameter volume coil for radio frequency transmission (Rapid Biomedical) and a 2-channel array surface coil (Rapid Biomedical) for signal reception.

The animals were anaesthetised with isoflurane (4–5% in O_2_) and placed in the scanner bore. Medetomidine (0.4 mg kg^−1^ bolus, followed by infusion 0.8 mg kg^−1^ h^−1^, s.c.) was administered to induce and maintain deep sedation for the duration of the experiment. Isoflurane was discontinued and the mice were free breathing with body temperature maintained at 37 ± 1℃ using a servo-controlled heating blanket. A nose cone was used to deliver oxygen enriched (∼30%) air and to apply CO_2_ challenges. To stimulate visual sensory pathways, 2 Hz pulses of cold white light were delivered to the scanner bore so that light reflected off the surface of the head coil stimulated the eyes with diffuse light. The stimulus was delivered using a block design paradigm of 40 s rest, 20 s activation, repeated three times.

Arterial spin labelling (ASL) was used to measure CBF at rest and during systemic hypercapnia. Baseline cerebral perfusion in the cortex was mapped using a flow sensitive alternating inversion recovery sequence with a three shot segmented gradient echo EPI readout (TE = 5.8 ms, TR = 5 s, TI = 2 s, 3 slices, 1 mm slice thickness). Repeated ASL images (acquired every 30 s) were captured for 10 min at baseline conditions, during 5 min of CO_2_ challenge (5% CO_2_ in the inspired gas mixture), and during a 3.5 min period of recovery. This experimental protocol was repeated three times for each the animal. Maps of CBF were generated by fitting the data to the established model, as described previously.^19^ The mean CBF within the cortex was plotted from manually drawn regions of interest (ROI).

The functional MRI (fMRI) methods used in this study were described in detail previously.^19,20^ Briefly, anatomical reference scans were acquired using a fast spin echo sequence (TR/TE_eff_ = 4000/48 ms, ETL = 8, matrix size = 192 ×192, FOV = 35 × 35 mm^2^, 35 coronal slices each 0.6 mm thick). Functional data were acquired using four snapshot GE-EPI sequence (FOV = 35 × 35 mm^2^, matrix size = 96 × 96, 12 coronal slices each 0.5 mm thick, slice gap 0.1 mm, spectral width = 178.6 kHz, TR = 2.5 s, TE = 19 ms, 131 volumes including one triple reference scan, total scan time approximately 5.5 min). Each subject underwent one anatomical reference scan and two fMRI scans.

**Figure 1.**
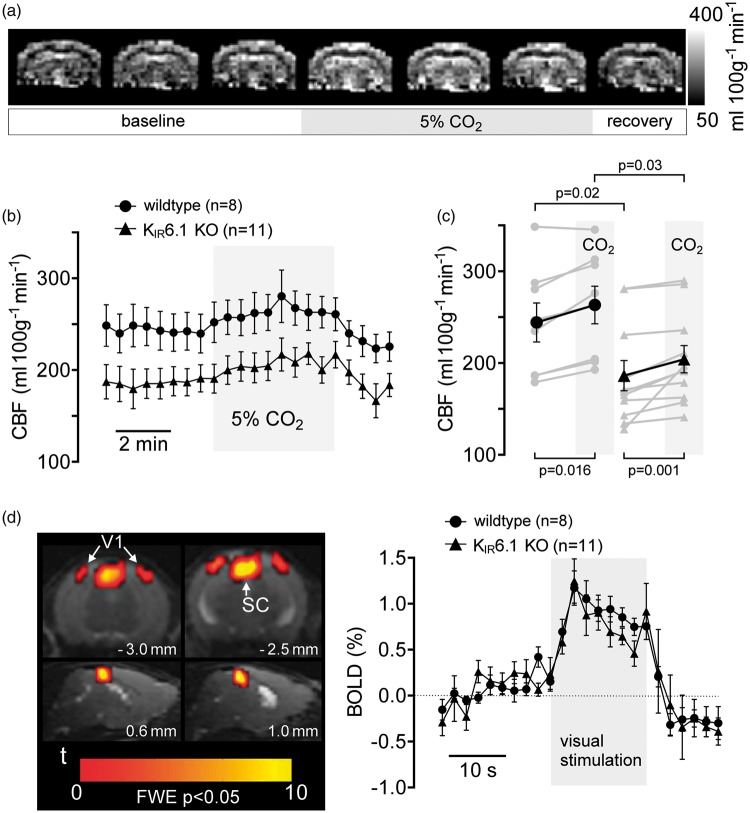
Resting cerebral blood flow (CBF), cerebrovascular reactivity to CO_2_, and blood oxygen level dependent (BOLD) fMRI responses in the visual cortex in mice lacking K_ATP_ channel subunit K_IR_6.1. (a) Representative arterial spin labelling brain maps illustrating measurements of CBF at baseline, during CO_2_ challenge (5% CO_2_ in the inspired gas mixture) and following recovery in a K_IR_6.1 knockout (KO) mouse (7 CBF maps of the total time series of 22 are shown); (b) Mean time-course of the whole brain CBF determined using arterial spin labelling MRI in K_IR_6.1 knockout and wildtype mice at resting conditions and in response to CO_2_ challenge; (c) Summary data illustrating resting CBF and peak increases in CBF in response to CO_2_ in K_IR_6.1 deficient and wildtype mice; (d) Representative BOLD activation maps (FWE, familywise error, *P* < 0.05, nv = 3) taken at two coronal (top, distance from Bregma is indicated) and two sagittal (bottom, distance from the midline is indicated) levels showing activation of visual pathways in the brain of a K_IR_6.1 knockout mouse and BOLD response curves illustrating changes in mean signal within the primary visual cortex (V1) induced by visual stimulation (20 s) in K_IR_6.1 knockout and wildtype mice. SC: superior colliculus. Data are presented as individual values and/or means ± SEM.

Acquired fMRI data were analysed offline using NiftyReg,^21^ in-house MATLAB 2013a scripts and SPM12,^22^ as previously described.^20^ Brain anatomical reference images were registered to the Allen Mouse Brain Atlas^23^ using an affine registration via an MRI template. Each affine transformation matrix was then applied to the individual fMRI data to normalise each subject into the atlas space. The registration was evaluated by visual inspection using SPM12 and the Mouse Brain Atlas.^24^ After registration, the fMRI data were realigned, corrected for differences in slice timing and smoothed (Gaussian FWHM of two voxels). Scans were screened for artefacts (gross motion, Nyquist ghosting and signal drop-out due to B_0_ field inhomogeneities) by visual inspection performed by the investigator blinded to the nature of the experimental group. After screening, three subjects were excluded from the analysis (two K_IR_6.1 knockout and one wildtype mice), giving final group sizes indicated in Figure 1. ROI-based analysis was conducted by using MRI atlas labels to determine time courses using the MarsBaR toolbox. Bilateral ROIs were chosen for timecourse extraction within V1 of the mouse visual cortex (region code VISp in the Allen Mouse Brain Atlas). BOLD signals were calculated by subtracting the mean baseline value from the mean BOLD values acquired during each stimulation epoch. All experiments were performed blindly and all the analyses were done by individuals blinded to the nature of the experimental groups.

### *Brain parenchymal* PO_2_ measurements

Anaesthesia was induced and maintained with isoflurane (5% induction, 2–3% maintenance). Core temperature was kept at ∼37℃ using a heating blanket. Carotid artery was cannulated to record systemic arterial blood pressure. The animal was placed in a stereotaxic frame and the skull was exposed. A small hole was drilled in the parietal bone above the primary visual cortex (V1) using the following coordinates: 1.0 mm rostral to lambda and 2.5 mm lateral from the midline. The dura was punctured and an Oxylite™ optical oxygen sensor (Oxford Optronix) was lowered into the cortical tissue to a depth of ∼1 mm from the surface of the brain. The craniotomy was then sealed with a layer of petroleum jelly to prevent diffusion of ambient oxygen. Parenchymal *P*O_2_ sampling continued for 10 min until a stable reading was achieved. *P*O_2_, *P*CO_2_ and pH of the arterial blood were measured using a Siemens blood gas analyser (RapidLab 248). Data were analysed off-line using *Spike 2* software (Cambridge Electronic Design).

### Statistics

Differences in grouped mean data were tested for significance using Wilcoxon’s signed rank test or Mann–Whitney test, where appropriate. Differences with *p* < 0.05 were considered to be significant

## Results

ASL method was first used to quantify CBF in the wildtype and K_IR_6.1 deficient mice (Figure 1(a) to (c)). Resting CBF was found to be lower in K_IR_6.1 knockout animals compared to that recorded in their wildtype littermates (186 ± 16 vs. 244 ± 21 ml 100 g^−1 ^min^−1^; *p* = 0.02). However, CBF CO_2_ responses were not affected in conditions of K_IR_6.1 deficiency. In response to a CO_2_ challenge (5% inspired CO_2_; 5 min; increase in the arterial *P*CO_2_ from 44±3 to 66 ± 3 mmHg), CBF increased from 244 ± 21 to 263 ± 21 ml 100 g^−1 ^min^−1^ in wildtype animals (0.35% ΔCBF per mmHg arterial *P*CO_2_) and from 186 ± 16 to 203 ± 15 ml 100 g^−1 ^min^−1^ in K_IR_6.1 knockout mice (0.44% ΔCBF per mmHg arterial *P*CO_2_). Thus, although resting CBF was significantly lower in K_IR_6.1 knockout animals, there was no difference in the magnitude of CO_2_-induced cerebrovascular responses between the groups (CBF increased by 19 ± 5 vs. 18 ± 6 ml 100 g^−1 ^min^−1^ in the wildtype and K_IR_6.1 knockout mice, respectively; *p* = 0.438) (Figure 1(c)).

Sensory-evoked BOLD fMRI responses were next assessed in the primary visual cortex of K_IR_6.1 knockout mice and their wildtype counterparts (Figure 1(d)). The time course data show that the magnitude and profile of cortical BOLD responses triggered by visual stimuli were not affected in conditions of K_IR_6.1 deficiency. Peak BOLD responses were 1.2 ± 0.6 %Δ in the wildtype mice and 1.3 ± 0.4 %Δ in K_IR_6.1 knockout mice (Figure 1(d)).

**Figure 2.**
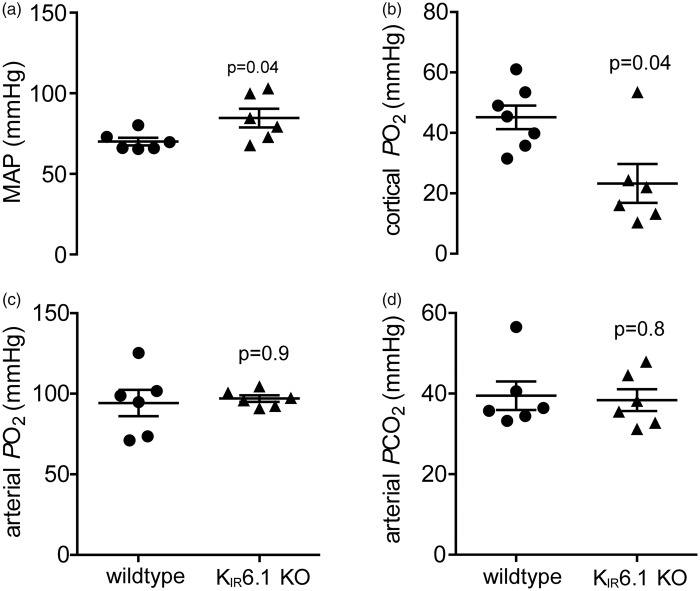
Reduced parenchymal partial pressure of oxygen (*P*O_2_) in the visual cortex in mice lacking K_ATP_ channel subunit K_IR_6.1. Summary data illustrating mean arterial blood pressure (MAP) (a) resting brain tissue *P*O_2_ (b), arterial *P*O_2_ (c), and arterial *P*CO_2_ (d) in K_IR_6.1 knockout (KO) and wildtype mice. Data are presented as individual values and means ± SEM.

Due to the recorded differences in resting CBF between the wildtype and K_IR_6.1 deficient mice (Figure 1(b) and (c)), we next determined whether reduced cerebral perfusion is associated with altered brain tissue *P*O_2_. Parenchymal *P*O_2_ was measured in the visual cortex in animals breathing room air under isoflurane anaesthesia. Mean systemic arterial blood pressure was found to be significantly higher in K_IR_6.1 knockout animals compared to that in wildtype mice (85 ± 6 vs. 70 ± 2 mmHg; *p* = 0.04) (Figure 2(a)), confirming data obtained in conscious K_IR_6.1 deficient mice.^11^ Although, the arterial blood pressure was higher, K_IR_6.1 knockout animals were found to have a significantly lower brain parenchymal *P*O_2_ than their wildtype counterparts (23.3 ± 6.4 vs. 45.1 ± 4.0 mmHg; *p* = 0.035) (Figure 2(b)). There were no differences in the arterial *P*O_2_, *P*CO_2_ and pH (7.34 ± 0.03, *n* = 6 vs. 7.38 ± 0.04, *n* = 6; *p* = 0.9) between K_IR_6.1 deficient and wildtype mice (Figure 2(c) to (d)).

## Discussion

In this study, we tested the hypothesis that K_ATP_ channels containing the K_IR_6.1 subunit (highly expressed by vascular smooth muscle cells and pericytes) maintain cerebrovascular tone and, therefore, play an important role in the control of CBF. The data obtained demonstrate that K_IR_6.1 deficiency in mice is associated with a significant reduction in basal CBF and brain tissue *P*O_2_, despite normal level of arterial oxygenation and higher systemic arterial blood pressure. Cerebrovascular reactivity to CO_2_ and sensory-evoked BOLD fMRI responses in the visual cortex (a measure of neurovascular coupling) were not affected in conditions of K_IR_6.1 deficiency.

Central to our original hypothesis, K_IR_6.1 channel activity can be modulated by various metabolic signals (such as increased energy demand or changes in pH) and, therefore, play a certain role in the mechanisms underlying cerebrovascular responses to increased neuronal activity.^25,26^ This hypothesis was supported by the evidence of strong K_IR_6.1 subunit expression in the arterial smooth muscle cells and brain pericytes.^13^ Despite high expression of K_IR_6.1 in contractile brain cells that regulate cerebrovascular tone and the molecular properties of K_IR_6.1 underlying detection and integration of metabolic signals, this channel appears to be dispensable for neurovascular coupling (as measured by the BOLD response).

Although K_IR_6.1 channel activity is sensitive to pH,^25^ K_IR_6.1 deletion had no effect on cerebrovascular reactivity to CO_2_, suggesting that this ATP and H^+^ sensitive channel is not involved in dynamic regulation of CBF. However, a significant limitation in interpretation of these results is that cerebrovascular responses to CO_2_ recorded in both K_IR_6.1 deficient and wildtype mice were markedly smaller compared to typical cerebrovascular CO_2_ reactivity of 2–4% ΔCBF change per mmHg of arterial *P*CO_2_ reported in other published studies, including data obtained by our group in isoflurane-anaesthetised mice.^27^ In the current study, ASL was performed under medetomidine sedation, which is the latest popular sedative for rodents in fMRI experiments. As hypercapnic challenge led to significant increases in the arterial *P*CO_2_, these data suggest that medetomidine may impair cerebrovascular reactivity. Although identical experimental protocols were applied to knockout and wildtype mice and similar absolute increases in CBF during hypercapnia in these two cohorts were recorded, we cannot exclude that K_ir_6.1 channels contribute to cerebrovascular CO_2_ reactivity in an unanaesthetized state or when studied using anaesthetics other than medetomidine.

Our data provide the first evidence that K_ATP_ channel activity is critically important for the control of basal CBF. K_IR_6.1 involvement in the control of brain perfusion is likely to be exerted at the level of cerebral supply vessels. This hypothesis is supported by the evidence that compounds which promote opening of K_ATP_ channels induce dilations of basilar and middle cerebral arteries.^12^ There is also evidence that pharmacological blockade of K_ATP_ channels can trigger constrictions of some cerebral vessels, including dural^28^ and middle meningeal arteries.^29^

An earlier study reported that in mice global K_IR_6.1 deficiency is leading to the development of systemic arterial hypertension,^11^ – the phenotype that is consistent with the role of this channel in determining peripheral vascular tone. However, there is evidence that brain hypoxia may contribute to the development of systemic hypertension by the recruitment of the brainstem hypoxia-sensitive mechanism, mediated by astrocytes,^30^ leading to enhanced central sympathetic drive.^31^ It is possible that reduced cerebrovascular flow and brain hypoxia observed in K_IR_6.1 deficient animals contribute to the development of hypertensive phenotype in this model.

In summary, the data obtained in the present study suggest that K_IR_6.1 is critically important for the maintenance of normal cerebral perfusion and brain tissue *P*O_2_, which ensures brain longevity. K_IR_6.1 appears to be dispensable for functional dynamic changes in CBF.
